# Mitochondria interaction networks show altered topological patterns in Parkinson’s disease

**DOI:** 10.1038/s41540-020-00156-4

**Published:** 2020-11-10

**Authors:** Massimiliano Zanin, Bruno F. R. Santos, Paul M. A. Antony, Clara Berenguer-Escuder, Simone B. Larsen, Zoé Hanss, Peter A. Barbuti, Aidos S. Baumuratov, Dajana Grossmann, Christophe M. Capelle, Joseph Weber, Rudi Balling, Markus Ollert, Rejko Krüger, Nico J. Diederich, Feng Q. HeFeng

**Affiliations:** 1 Instituto de Física Interdisciplinar y Sistemas Complejos IFISC (UIB-CSIC), E-07122 Palma de Mallorca, Spain; 2 Center for Biomedical Technology, Universidad Politécnica de Madrid, Campus of Montegancedo, E-28223 Pozuelo de Alarcón, Madrid Spain; 3 Luxembourg Centre for Systems Biomedicine (LCSB), University of Luxembourg, Campus Belval, 6, Avenue du Swing, L-4367 Belvaux, Luxembourg; 4 Transversal Translational Medicine, Luxembourg Institute of Health (LIH), 1A-B, rue Thomas Edison, L-1445 Strassen, Luxembourg; 5 Disease Modeling and Screening Platform (DMSP), Luxembourg Institute of Systems Biomedicine, University of Luxembourg & Luxembourg Institute of Health, 6 avenue du Swing, L-4367, Belvaux, Luxembourg; 6 Department of Infection and Immunity, Luxembourg Institute of Health (LIH), 29, rue Henri Koch, L-4354 Esch-sur-Alzette, Luxembourg; 7 Centre Hospitalier de Luxembourg (CHL) 4, Rue Nicolas Ernest Barblé, L-1210 Luxembourg, Luxembourg; 8 Department of Dermatology and Allergy Center, Odense Research Center for Anaphylaxis (ORCA), University of Southern Denmark, 5000C Odense, Denmark; 9 Institute of Medical Microbiology, University Hospital Essen, University Duisburg-Essen, D-45122 Essen, Germany

**Keywords:** Systems biology, Neurology, Systems analysis, Diseases

## Abstract

Mitochondrial dysfunction is linked to pathogenesis of Parkinson’s disease (PD). However, individual mitochondria-based analyses do not show a uniform feature in PD patients. Since mitochondria interact with each other, we hypothesize that PD-related features might exist in topological patterns of mitochondria interaction networks (MINs). Here we show that MINs formed nonclassical scale-free supernetworks in colonic ganglia both from healthy controls and PD patients; however, altered network topological patterns were observed in PD patients. These patterns were highly correlated with PD clinical scores and a machine-learning approach based on the MIN features alone accurately distinguished between patients and controls with an area-under-curve value of 0.989. The MINs of midbrain dopaminergic neurons (mDANs) derived from several genetic PD patients also displayed specific changes. CRISPR/CAS9-based genome correction of alpha-synuclein point mutations reversed the changes in MINs of mDANs. Our organelle-interaction network analysis opens another critical dimension for a deeper characterization of various complex diseases with mitochondrial dysregulation.

## Introduction

Network-biology approaches are successfully employed for a better understanding of complex diseases that are caused through interactions between genetic and/or environmental factors^1–6^. Small- and macromolecules such as genes, proteins, and/or metabolites interact with each other and form networks with certain common underlying organization principles, in sharp contrast to random networks. All these molecular networks seem to obey to a general scale-free power-law distribution principle^7^, although the definition of power-law distribution might require fine adjustment^8^. Mitochondria, the key organelles regulating cellular metabolism and generating cellular energy, constantly interact with each other, i.e., via the fusion and fission processes. Therefore, they form perpetually changing networks. Nevertheless, it remains unclear whether such organelle interactions form random networks, or in contrast well-organized structures obeying universal principles. Answering this question could open basic new research avenues in neurodegeneration as mitochondrial dysfunction is connected to several neurodegenerative diseases, such as Huntington’s disease, Alzheimer’s disease, and Parkinson’s disease (PD)^9–12^. Therefore, we here took advantage of the availability of various PD-derived tissues and analyzed in all of them whether a functional impairment of mitochondria is associated with any specific topological patterns or features of large-scale mitochondria interaction networks (MINs).

## Results

To obtain more precise information on mitochondria interactions, we used high-resolution 3D mitochondrial immunofluorescence images in the ganglia from the ascending (left) and descending (right) colon collected from idiopathic PD patients and healthy controls^13^. We extracted network adjacency matrices of mitochondria interactions from all ganglia neurons in such a way that mitochondria branch points were represented as nodes, with an undirected link being present if an interaction is observed between a pair of nodes at the imaging moment. We performed various types of network analyses (up to 19 different network structure metrics, refer to “Methods”) in this work. In the second step, the same network analysis was applied to midbrain dopaminergic neurons (mDANs) differentiated from induced pluripotent stem cells (iPSCs) derived from skin fibroblasts of genetic PD patients and the corresponding healthy controls. We analyzed the MINs in the samples from patients with heterozygous point mutations, namely in the *SNCA* gene (*PARK1*) encoding alpha-synuclein^14^, in the PD-associated gene *RHOT1* encoding a mitochondrial outer membrane GTPase^15,16^ (MIRO1), and in the *VPS35* gene (*PARK17*) encoding the vacuolar protein sorting-associated protein 35 (VPS35)^17–19^. We compared the samples of patients with those from age- and gender-matched healthy controls, in addition to the mutation-corrected isogenic controls from the patient harboring the heterozygous *SNCA* mutation^20^ (for patient information, refer to “Methods” and Supplementary Table 1). Furthermore, considering the existence of genetic background heterogeneity between often-used groups of cases and controls, we also compared one early-onset PD patient with another form of *SNCA* genetic modification, i.e., *SNCA* triplication^21^ and the first-degree consanguineous healthy control. For the mDANs derived from *VPS35*-mutated samples, we also analyzed under different physiologically and/or pathologically relevant culture conditions (i.e., with or without antioxidants). In total, here we analyzed MINs from 15 PD patients versus (vs.) 10 healthy controls (Supplementary Table 1).

### MINs in enteric ganglia neurons form nonclassical scale-free supernetworks and are composed of larger subnetworks in PD

**Fig. 1:**
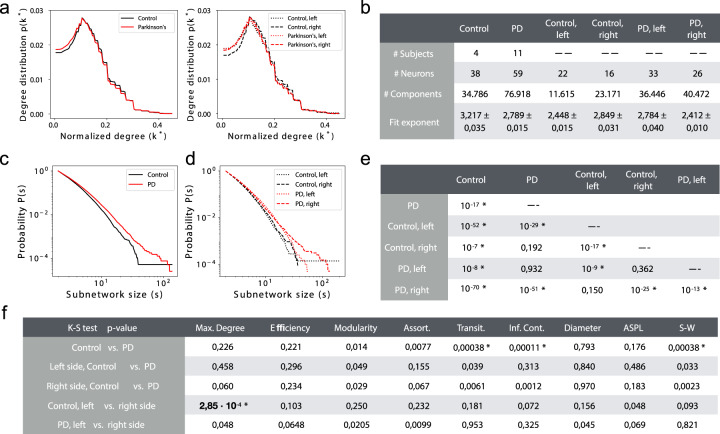
Topological properties of mitochondria interaction networks (MINs) from enteric ganglia of PD. **a** Probability distribution of the normalized degree of nodes within MINs of PD or healthy controls or indicated subgroups of samples. The displayed degree was normalized by the number of nodes in the given network component. **b** Summary of the network components of various sets of patients’ materials. A dashed line in the element indicates no entry. **c**, **d** Cumulative distribution of the component/subnetwork size of the MINs among all samples of healthy controls and PD patients (**c**), and among subsets of samples taken from the left or right side (**d**). **e**, **f** *P* value of the test (see “Methods“) evaluating the null hypothesis that the exponential fits of the degree distribution for the given two groups share the same power-law slope *k* (**e**), or evaluating through a two-sided K–S (Kolmogorov–Smirnov) test the null hypothesis that the distribution of each topological metric of the global MINs is identical for the given two groups (**f**). In **e**, only the lower triangular matrix of all the pairwise comparisons is displayed for simplification. Asterisk indicates a significant *P* value < =0.05 after Šidák correction (the displayed *P* values are before correction). PD patients with Parkinson’s disease, Control healthy controls, Assort. assortativity, Transit. transitivity, Inf.Cont. information content, ASPL average shortest pathway length, S-W small worldness.

As we hypothesized that the universal scale-free principle^22^ would also apply to mitochondria MIN, we first analyzed whether mitochondria form such a network within ganglia. We found that in the MINs, the probability *p(k)* that a node interacts with *k* other nodes did not follow a power law^7^ (i.e., *p(k)~k*^*ɣ*^) (Fig. 1a). This result indicates that MINs did not self-organize into standard scale-free networks. The inability of MINs to form scale-free networks was independent from the subject groups (PD patients or healthy controls) and from the sample origins (left- or right-side biopsies) (Fig. 1a). Within the ganglia, the mitochondria formed various sizes of connected subnetworks/components (>16 thousands of subnetworks per group) with different numbers of mitochondria branch points (nodes) and interaction structures (Fig. 1b). We therefore checked whether the size of these subnetworks, which are completely disconnected from one another and thus represent independent mitochondrial network structures, is organized according to a scale-free principle. Interestingly, the overall mitochondria interactions formed a nonclassic modular scale-free network, where the probability *P(s)* that one subnetwork with at least *s* nodes exists indeed decays as a power law, following *P(s)~k*^*s*^ (Fig. 1c, d). Unexpectedly, however, the MINs from PD patients were more frequently composed of larger subnetworks than the MINs from healthy controls (*P* value = 10^−17^, see “Methods,” Fig. 1c–e). This difference between healthy controls and PD patients was more evident in the MINs from ganglia derived from the right/ascending colon biopsies than that from the left/descending colon (*P* value = 10^−25^, Fig. 1d, e), possibly due to the assumed rostrocaudal disease progression within the gastrointestinal tract^23^.

### Alteration in network topological features of MINs from enteric ganglia neurons of PD

**Fig. 2:**
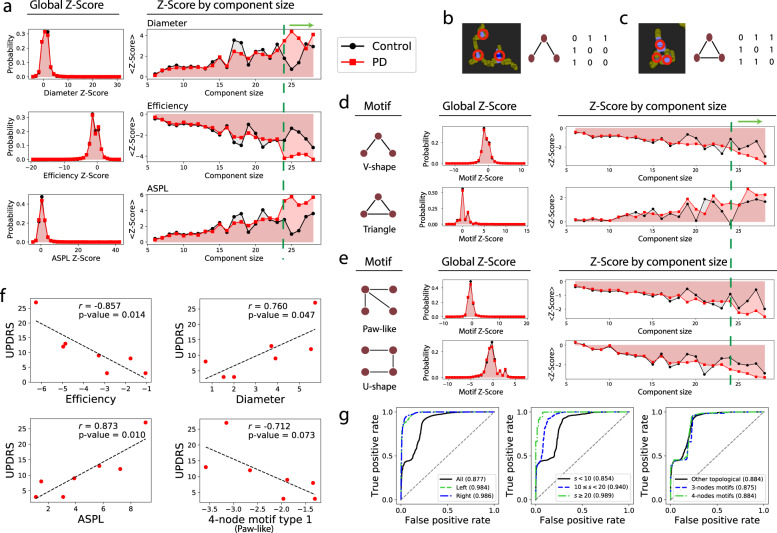
PD MINs show significantly altered motifs and network features and can be used for accurate sample classification. **a** Distribution of *Z* scores of different network metrics. Left, histograms representing the probability distribution of the corresponding *Z-* score from the global MINs. The right graphs depicting the *Z* scores for the given size of the MIN components/subnetworks. The green dashed line and the green arrow above the plots highlight the large components. **b**, **c** Example of partially- (**b**) or fully (**c**) connected 3-node MIN motifs. Left, representative mitochondrial immunofluorescence images; middle, the diagram of the undirected network motif; right, the corresponding adjacency matrix (the pixel values in the link were simplified to 1 if there is a link and otherwise 0). Red circles in the left panel indicate where the nodes are positioned. **d**, **e** Distribution of the two types of 3-node (**d**) or 4-node (**e**) motifs from PD patients and healthy controls. Left, the diagram of the corresponding motif; Middle, the histograms representing the probability distribution of the corresponding *Z-* scores from healthy controls (black bar) or PD patients (red bar); the right graphs depicting the *Z* scores for various sizes of the network components. Since the component with 29 nodes only appeared once in the healthy controls, we did not include very large components (size > = 29) in the analysis. Furthermore, for all the control subjects and PD patients, only 9 and 46 components with the size > = 29 existed in the MINs, representing only 0.0471% and 0.115% of the total number of components, respectively. **f** Correlation analysis between individual patient UPDRS clinical score and network efficiency, diameter, average shortest path length (ASPL), and paw-like 4-node motif (the first one in **e**) within the network components with the size of 28. The parameter r is the Pearson correlation coefficient. *P* value is the probability that the correlation coefficient is in fact zero. Of note, not all the patients have the corresponding component (size of 28). **g** Accurate classification of PD patients from healthy controls using the MIN topological features alone. Each graph depicts multiple ROC curves and the corresponding area under ROC curve (AUC). Left, central, and right panels respectively present classification results using samples from left- or right-side or both-side ganglia, from components with different sizes, and from various indicated features.

To systematically explore the topological properties of the MINs, we calculated various other topological/graphic metrics^24^, such as network max degree, diameter, efficiency, average shortest path length (ASPL), modularity, assortativity, transitivity, information content, and small worldness (for the definition of different network property metrics, refer to “Methods”)^25^. We first focused on three network metrics with obvious biological meaning and implications. Those parameters, i.e., network diameter, efficiency, and ASPL represent one group of closely related metrics, which all essentially signify how efficiently the energy and information can be transferred and distributed among different nodes/mitochondria within enteric ganglia neurons. The larger the ASPL within a given MIN subnetwork, the less efficiently the MIN subnetwork transfers the energy from one node to another one. Among various analyzed metrics, we only observed marginal differences for transitivity (demonstrating density of triangles), information content (assessing the presence of regular mesoscale structures)^26^, and small worldness (S-W) between the global MINs from PD patients compared to healthy controls (Fig. 1f and Supplementary Fig. 1). As no topological difference was substantial in the mesoscale properties, we searched for network feature differences at a microscale level. Interestingly, for the components with the number of nodes equal to or larger than (≥) 24, we noticed that the average *Z* scores of the efficiency were much lower in PD than in healthy controls, whereas those of ASPL were much larger in PD (Fig. 2a, right panel). In line with this notion, the normalized network diameter for the larger components was much larger in PD than in healthy controls (Fig. 2a, right panel). It is worthy noting that the distribution peak of network diameter, efficiency, and ASPL as shown in the histograms, no matter within the MINs of controls or PD patients, was very narrow (Fig. 2a, left panel), indicating the homogeneity of those features among different subjects. To partially circumvent the issue of the imbalanced numbers between the controls and the PD patients, we iteratively removed one subject in each group. Interestingly, the resultant variation of those metrics in PD and controls was very low, and the difference in those topological indexes between PD and controls was clearly maintained (Supplementary Fig. 2a). Furthermore, we randomly selected four out of the eleven PD patients and repeated this process 100 times, which made the size of the PD patient and the control group comparable. Notably, the resulting variation in those tested network features was still very small, further indicating the homogeneity of those indexes in the MINs. Therefore, the imbalanced number of subjects recruited in the comparison groups did not affect our conclusion in this study (Supplementary Fig. 2b). These results may explain why PD patients have dysfunctional ganglia neurons^27^. The observed lower network efficiency and accordingly increased ASPL in MINs might have important implications, i.e., energy and information within enteric ganglia neurons are possibly produced, shared, and distributed less competently in the ganglia neurons of PD patients relative to healthy controls. On the other hand, similar to that of the power grid^28^, these network topological features of the MINs may also serve as a protective compensatory mechanism in PD patients. More investigation is required to distinguish between primary pathogenic and secondary compensatory mechanisms.

Network motifs are recurrent conserved building blocks composed of a small number of nodes that are often associated with certain functions^29^. Without consideration of the network component size, there was no clear difference in the *Z* scores of various types of analyzed motifs between PD patients and healthy controls (for *Z* score, see “Methods,” Fig. 2b–e, Supplementary Fig. 3). Notably, for the components with the number of nodes ≥24, we noticed that the partially connected V-shape 3-node motifs existed less frequently in PD patients than in healthy controls (Fig. 2d). This observation seemed to be generally applicable, as it also held true for the partially connected paw-like 4-node motifs (Fig. 2e). We also checked other types of MIN motifs and found that the fully connected triangle 3-node motifs possessed a much higher *Z* score in PD than in healthy controls for large components (size > = 24, Fig. 2d). This observation was not evident for more-connected 4-node motifs possibly because of the decreased overall frequency of such complex motifs in MINs and randomized networks (Supplementary Fig. 3). In the mitochondria-interacting “social” networks, “dysfunctional” mitochondria of PD patients relative to “normal” mitochondria of controls might need to fully interact with each other in order to guarantee the necessary cellular energy supply. Higher frequency of this type of motifs may also partially compensate for the substantial right-side ganglionic shrinking in PD patients^13^. It is noteworthy that the fully connected triangle 3-node motifs, as those analyzed in the index of transitivity, are the most recurring motifs in many different types of biological and social networks^30,31^, reflecting the relevance and importance of such types of motifs in establishing network efficiency and maintaining network functions.

### Network topological features are correlated with PD clinical scores

With these promising results in terms of difference in topological patterns of the MINs from macro- to meso- to microscale levels in mind, we explored whether some of these network features are correlated with the most-used clinical scores, i.e., Unified Parkinson’s Disease Rating Scale (UPDRS)^32^. If correlated, those features could be used as potential state markers of the early PD phase, with the potential for application in trials with PD disease-modifying treatments. Notably, network efficiency is significantly negatively correlated with the UPDRS scores (*r* = −0.857, *P* value = 0.014) for a large size of network components (size of 28; this size exists among different individuals), indicating that a lower network efficiency reflects more severe PD motor symptoms in individual patients (Fig. 2f). Concordantly, as the related but approximately inverse parameters of network efficiency, ASPL and diameter are positively correlated with the UPDRS scores for the corresponding components (size of 28) (Fig. 2f). The *Z* scores of the paw-like 4-node network motifs were also negatively correlated with the UPDRS scores, although to a lesser extent (Fig. 2f).

### Network topological features of MINs alone can accurately discriminate PD patients from controls

Having found such a high correlation between network features and well-accepted PD clinical scores, we applied machine-learning approaches to assess whether we can use a combinatory panel of those network features as more powerful biomarkers. After testing and comparing several algorithms in both our real and randomized datasets with reshuffled sample labels (Supplementary Fig. 4), we selected a high-performance machine-learning approach (i.e., multilayer perception (MLP)) with leave-one-out cross-validation to discriminate the samples of PD patients from healthy controls (Fig. 2g). When only choosing features from right- or left-side ganglia, we found that the area under the ROC curve (AUC), the essential performance index of biomarkers, was as high as 98.6% and 98.4%, respectively (Fig. 2g, left panel). When we mixed the features from both right- and left-side samples, the AUC was still maintained at 87.7% (Fig. 2g, left panel). The classification results using various sizes of components of MINs showed that PD-specific features were mainly encoded in large subnetworks (AUC = 98.9% for size > = 20, Fig. 2g, middle). Integration of different types of key topological features is necessary to reach accurate classification (Fig. 2g). Together, these results demonstrate that the features in the MINs represent very valuable information and can be used as potent novel biomarkers for the PD diagnosis.

### MINs within dopaminergic neurons derived from genetic PD patients also show altered network features

**Fig. 3:**
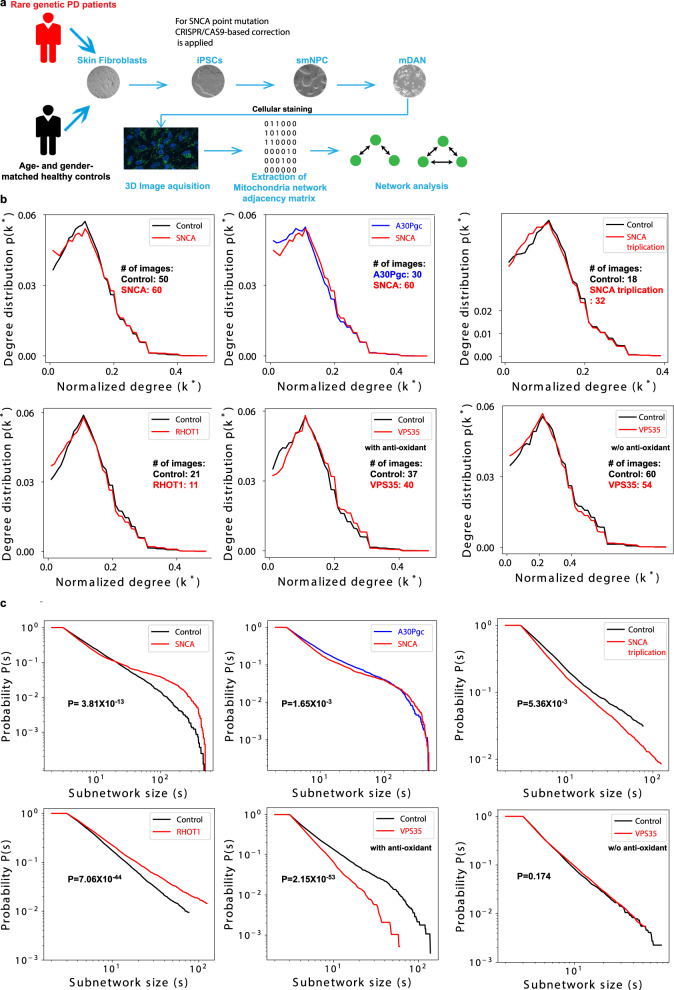
MINs within iPSC-differentiated dopaminergic neurons of certain genetic PD patients show similar alterations as those in ganglia of sporadic PD patients. **a** Schematic on how to perform network analysis from dopaminergic neurons differentiated and derived from skin fibroblasts of human subjects. Details are provided in “Methods”. Here, we only described briefly. iPSCs were first reprogrammed from skin fibroblasts. Using small molecules, we then differentiated iPSCs to small-molecule neural precursor cells (smNPCs). Finally, using trophic factors, smNPCs were differentiated to midbrain dopaminergic neurons (mDANs). We then performed cellular staining to identify mitochondria within each mDAN and identified mitochondria–mitochondria interactions using confocal 3D mitochondrial immunofluorescence images. After extracting network adjacency matrices, we then performed large-scale network analysis on the MINs. **b**, Probability distribution of the normalized degree of nodes within MINs of iPSC-differentiated mDANs derived from skin fibroblasts of different genetic PD patients or the corresponding age- and gender-matched healthy controls (refer to “Methods”) or *SNCA*-mutation-corrected lines using the CRISPR/CAS9 approach. Of note, mDANs from the *VPS35*-mutated patient and the matched controls were differentiated with or without (w/o) antioxidants (as indicated), while the others were all differentiated in the presence of antioxidants. **c** Cumulative distribution of the component/subnetwork size of the MINs among all the samples of different patients or controls or patients’ isogenic controls. Displayed *P* value of the test (see “Methods”) evaluating the null hypothesis that the exponential fits of the degree distribution for the indicated two groups of the given plot share the same power-law slope *k*.

To further check whether our observation in enteric neurons of idiopathic PD patients holds true in mDANs derived from genetic PD patients, we generated human iPSC-differentiated mDANs and analyzed their MINs (Fig. 3a). Again, the MINs in iPSC-differentiated mDANs, no matter being derived from which genetic PD patients or age- and gender-matched healthy controls, did not self-organize into standard scale-free networks similar as observed in enteric ganglia neurons (Fig. 3b). In line with the observation in enteric ganglia, the MINs of iPSC-differentiated mDANs derived from a PD patient with a point mutation in the *SNCA* gene that leads to an A30P amino acid exchange in the encoded protein also formed a nonclassic scale-free supernetwork (Fig. 3c). Consistent with the effect on subnetwork sizes of the MINs from the idiopathic PD patients, the *SNCA*-mutated patient showed much larger subnetworks than those from the healthy controls (50 or 60 different measurements or clones per group, *P* value = 3.81 × 10^−13^, Fig. 3c). This also holds true for that from one *RHOT1*-mutated patient relative to the age- and gender-matched healthy control (*P* value = 7.06 × 10^−44^). However, the correction of the point mutation in *SNCA* using CRISPR/CAS9-based genome editing did not dramatically, although still significantly (*P* value = 1.65 × 10^−3^), enhance the size of the subnetworks (Fig. 3c). In contrast to the observations of other mutations, the *VPS35*-mutated patient showed substantially smaller subnetworks than the matched healthy controls (*P* value = 2.15 × 10^−53^, Fig. 3c), which is in line with the reported observation that *VPS35* mutations cause the fragmentation of individual mitochondria^18^. To further test whether the effect of the *VPS35* mutations is regulated by oxidative stress, we exposed the cells to oxidative stress during the iPSC-differentiation process. Following oxidative stress, the difference disappeared in subnetwork size of MINs within mDANs derived from the *VPS35*-mutated patient vs. the matched control, indicative of the direct involvement of oxidative stress in *VPS35*-mediated feature changes of the MINs. Alike to what was seen in the MINs of *VPS35*-mutated samples cultured with antioxidants, a decrease was observed in subnetwork size of the MINs of mDANs derived from the patient with *SNCA* triplication vs. the gender-matched unaffected immediate family member (Fig. 3c). The distinction observed between the *VPS35*-mutated samples and the other tested samples with point mutations might be simply attributable to the fact that the D620N mutation in *VPS35* disrupts both the distribution of endosomes^33^ and mitochondrial functions^18^, while the other PD genetic point mutations mainly affect the latter. Although both forms of *SNCA* genetic variants, e.g., the point mutations in *SNCA* and *SNCA* triplication, show bioenergetic dysfunction in derived mDANs^34^, their effects on MIN subnetwork size were opposite (Fig. 3c). Therefore, the effect on subnetwork size of MINs of mDANs might be dependent on several aspects, such as which familial PD gene is mutated, how the genetic variants are modified, and whether the given mutation directly contributes to mitochondrial dysfunction and is regulated by oxidative stress.

**Fig. 4:**
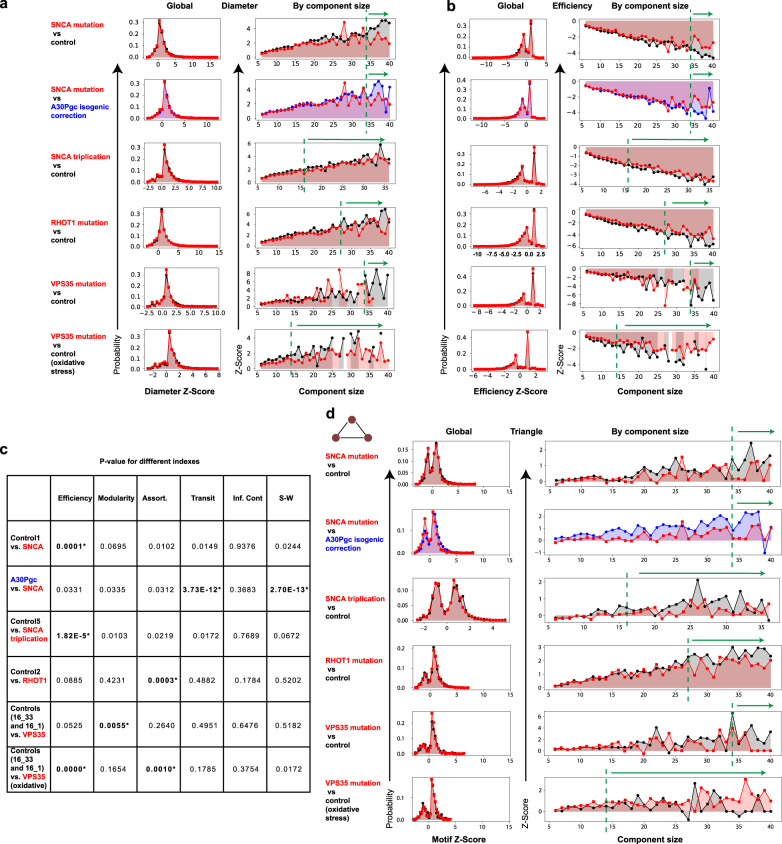
MINs of iPSC-differentiated mDANs of different genetic PD patients show consistent alteration in network features. **a**, **b** Distribution of *Z* scores of different network structure features, for instance network diameter (**a**) and efficiency (**b**). Left, histograms representing the probability distribution of the corresponding *Z* score from the global MINs of mDANs derived from different patients or matched controls; the right graphs depicting the *Z* scores for the given size of the MIN components/subnetworks. The green dashed line and the green arrow above the plots highlight the large components that show a clear difference. Since the component with 37 nodes only appeared once in the corresponding healthy controls matching the *SNCA*-triplication patient, we did not show very large components (size > = 37) in the analysis. **c** *P* values of the two-sample two-sided K–S (Kolmogorov–Smirnov) test evaluating the null hypothesis that the distribution of each indicated topological metric of the global MINs is identical for the given two groups. Asterisk indicates a significant *P* value < = 0.05 after Šidák correction (the displayed *P* values are before correction). **d** Distribution of the fully connected 3-node MIN motifs (known as “triangle”) of mDANs derived from different patients or matched controls; Left, the histograms representing the probability distribution of the corresponding *Z* scores of global MINs from different genetic PD patients or matched controls; the right graphs depicting the *Z* scores for various sizes of the network components.

To obtain a more comprehensive understanding of the network features of MINs, we investigated and compared other network topological indexes of the MINs from mDANs derived from genetic PD patients. Keeping in mind the observations in colonic ganglia, we particularly checked the topological metrics related to network efficiency. Notably, the MINs from the three rare genetic PD patients all presented smaller diameters for the subnetworks that are composed of nodes larger than a certain number (≥34, 16, 27, 34, and 14 for *SNCA* mutation, *SNCA* triplication, *RHOT1* mutation, and *VPS35* mutation with or without oxidative stress, respectively, Fig. 4a), whereas the efficiency was always higher than that of age- and gender-matched healthy controls (Fig. 4b). Interestingly, although *SNCA* triplication also causes bioenergetic dysfunction in mDNAs^34^, compared with influence of the *SNCA* A30P-point mutation on network diameter and efficiency, *SNCA* triplication showed still similar, but less profound effects on those network metrics (Fig. 4a, b). Correction in the *SNCA* mutation reversed both changes in network diameters and efficiency caused by the *SNCA*-point mutation (Fig. 4a, b). As determined by the definition of ASPL, the change of the ASPL in genetic PD patients is correlated with that of network diameter (Supplementary Fig. 5a). Of note, again the effect of the *VPS35* mutation on these MIN indexes, when the differentiation was performed with antioxidants, was smaller compared with that of the other analyzed genetic factors in this work. As oxidative stress worsens the iPSC-derived mDAN phenotypes of several genetic factors that contribute to the pathogenesis of PD^35,36^, the influence of the *VPS35* mutation under oxidative stress on particular network indexes became more evident (Fig. 4a, b and Supplementary Fig. 5a). In short, closely associated network indexes analyzed here, such as diameter, efficiency, and ASPL, showed consistent alteration in all the selected genetic PD patients. The results of the three network parameters demonstrated that the MINs within mDANs derived from several genetic PD patients all have enhanced efficiency in terms of energy transfer among different mitochondria within those larger subnetworks. Most likely, these consistent alterations in particular network features represent a conservative compensatory mechanism that tends to protect mDANs of tested genetic PD patients from death.

We further analyzed other network topological indexes of MINs in mDANs that were also calculated in enteric ganglia neurons of idiopathic PD patients. Interestingly, correction in the *SNCA* mutation significantly affected network transitivity and small worldness (Fig. 4c, for transitivity and small worldness, refer to “Methods” for the definition). The MINs of mDANs derived from both *VPS35*-mutated materials under oxidative stress and *RHOT1*-mutated patient samples showed significantly changed assortativity, a network metric representing to which extent highly connected nodes in a network tend to link with each other^37^ (Fig. 4c, refer to “Methods”). Furthermore, the MINs of mDANs derived from *VPS35*-mutated materials under oxidative stress, *SNCA*-point-mutated, and triplication materials all showed a significant change in the MIN network efficiency even on a global scale (Fig. 4c) in addition to those visible only in larger subnetworks (Fig. 4b). For the mDANs derived from the *VPS35*-mutated patient materials cultured with antioxidants, only modularity of the global MINs that measures how much the network is organized into communities showed a significant difference (Fig. 4c). Thus, alike to the effect on MIN subnetwork size, the influence on particular network properties is also dependent on specific genetic mutations and is affected by exogenous oxidative stress.

**Fig. 5:**
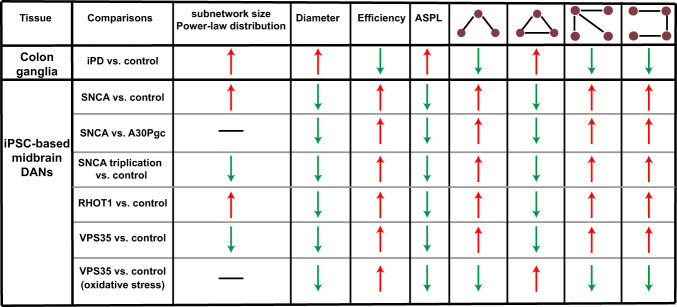
Summary of different network metrics of MINs analyzed in different types of PD samples. Red upward arrow indicates an enhanced feature, while the green downward arrow designates a reduction in the given network feature. Horizontal line indicates no clear change between the compared groups. iPD idiopathic PD, ASPL average shortest path length.

We further examined the 3- and 4-node motifs of MINs from those genetic PD patients. Interestingly, the triangle motifs, the most abundant network motifs in different types of networks^30,31^, within the mDANs differentiated with antioxidants, also displayed similar changes for larger MIN subnetworks of all patients carrying *SNCA*- or *RHOT1*- or *VPS35*-point mutations or *SNCA* triplication (Fig. 4d). Since both cellular MINs and power/electricity grid networks^31^ might share similar functions in terms of “energy transferring,” we reasoned that the frequency reduction in the triangle motifs of the larger MIN subnetworks from those genetic PD patients might enhance the risk of energy-supply failure and eventually harm the functions and survival of those neurons. The correction in the *SNCA*-point mutation again reversed the frequency change of triangle motifs caused by the *SNCA* mutation (Fig. 4d). It is noteworthy that under oxidative stress the MINs derived from the *VPS35*-mutated patient showed an inverted change as that of mDANs derived from the PD patients with point mutations or copy number variants in any of the three analyzed key PD genes, when being differentiated in the presence of antioxidants. This oxidative-stress induced effect of the *VPS35* mutation on the frequency of triangle motifs of iPSC-differentiated mDANs was in fact similar to that observed in the ex vivo analysis of colonic ganglia neurons of idiopathic PD patients (Fig. 2d). These results are very much in line with the current well-established paradigm that oxidative stress plays a critical role in dopaminergic neurotoxicity^38^ (Fig. 4d). The frequency change of the V-shape 3-node motifs was similar, although to a lesser degree, as that of triangle motifs (Supplementary Fig. 5b). The frequency change of both paw-like and U-shape 4-node motifs in those genetic PD patients was still similar to that of the triangle 3-node motifs in larger MIN subnetworks (Supplementary Fig. 5c, d). Nevertheless, the altered degree between the genetic PD patients and the controls in the frequency of the analyzed 4-node motifs was smaller compared with that of the triangle motifs. In summary, the analyzed network motifs also showed consistent changes for five out of the six comparison groups/conditions among genetic PD (Fig. 5). The only exception existed in the MIN network motif features caused by the *VPS35* mutations that were imposed by oxidative stress (Fig. 5). Taken together, although not always identical in changes for a wide range of examined indexes, the image datasets from both idiopathic and genetic PD patients revealed novel critical changes in the network topological structure of MINs that are associated with PD.

## Discussion

Early attention has already been paid to changes in mitochondria clusters within cells following oxidative stress^39^. Recently, the identification and analysis of protein–protein interaction networks within mitochondria in human immortalized cell lines has been performed^40^. Although these studies have already put mitochondria under the “network” umbrella, their analysis has still been focused on individual mitochondria levels, either on mitochondria organelle as a whole or on clusters of mitochondria. Since mitochondria constantly interact with each other and most likely do not work alone, it is rational to analyze the mitochondria interaction networks (MINs). However, such an analysis has never been performed even in general populations, not to mention among the patients with neurodegenerative diseases with direct mitochondrial involvement. As many molecular networks share certain underlying organizing principles, we aimed to investigate whether the organelle interaction networks, e.g., MINs follow similar principles, and whether and how the network topological properties are affected in relevant pathological conditions that are related to mitochondrial deficiency. To this end, we here comprehensively analyzed a variety of network topological indexes of MINs, contrary to a conventional analysis focusing on individual mitochondria-based phenotypes such as mitochondrial number, volume, size, shape, and even simplified network-like analysis still only on connection degree^13,41^. Beyond the intended proof-of-principle analysis, we found remarkable pattern differences in the MINs of enteric ganglia of sporadic PD patients vs. healthy controls. Furthermore, particular network metrics were highly correlated with PD clinical scores, indicating a potential of using particular network features for early diagnosis and basic research purposes of PD. Excitingly, with network topological features alone, we can already accurately distinguish the PD patients from healthy controls. This discovery opens a door to a new type of biomarkers from the perspective of network structure features of MINs in patient-based materials. However, further validation in a large-scale cohort or even multicenter cohorts is required. In PD patients, these differences in MINs might be directly related to well-known mitochondrial complex I deficiency^42^, mitochondrial fragmentation, and/or deficient mitochondrial dynamics^43,44^. In this work, we demonstrated the association between altered network topological indexes of MINs with known mitochondrial deficiency of sporadic PD patients.

Network analysis of MINs of mDANs derived from all the tested genetic PD patients vs. age- and gender-matched healthy controls revealed consistent changes for several related network metrics, such as diameter, efficiency, and ASPL. Remarkably, the change of direction seen in genetic PD patients vs. controls is in sharp contrast to that seen in sporadic PD patients. The difference in change direction of particular network features might be caused by several factors: (1) genetic PD vs. idiopathic PD, (2) tissue difference (enteric neurons vs. mDANs), (3) ex vivo imaging in ganglia vs. imaging on in vitro-derived cells, and (4) also possibly direct disease involvement vs. secondary compensation mechanism. Therefore, the inverted direction of change is plausible due to these fundamental differences. Importantly, despite a huge difference in the roles of the tested genetic factors, the consistency in pattern changes of particular network indexes (e.g., network efficiency-related indexes and triangle motifs) among different genetic PD patients underscores the value of using this type of MIN network analysis to assist diagnosis and classification of genetic PD patients. Such a consistency in pathology among different genetic PD patients has so far not been reported in other studies without the application of such a fundamental network analysis in MINs. Machine-learning-based computational analysis of MINs provides another layer of new information and enables automatized classification of a large number of subjects.

We also noticed a general negative correlation between the changed directions (increase or decrease) in network efficiency and triangle motif frequency of PD patients, independent of samples from sporadic or genetic PD patients (Fig. 5). These two important network metrics might well compensate each other to fine-tune the overall functions of mitochondria networks in PD patients, no matter in which tissues we analyzed. Interestingly, the only exception existed in the MINs of the mDANs derived from the *VPS35*-mutated patient materials under oxidative stress. In that case, both network efficiency and triangle motif frequency in the MINs were simultaneously heightened, possibly to fight against or compensate the strong cytotoxic effects induced by oxidative stress.

Due to the limited access to colonic ganglia samples from healthy controls, we were unable to analyze more healthy controls at the current stage of the project. To generate a reliable resource as the reference patterns of different network topological metrics within MINs, a large-scale study is further required at different tissue levels and cell types, by enrolling healthy volunteers with different ethnicities, gender, and age groups. We also could not access the ganglia materials from patients with other types of diseases, in particular from other (e.g., inflammatory) colon diseases. Due to this lack of comparison with samples from non-PD patients, we cannot conclude whether our observed changes in the structural features of the MINs are PD-specific or not. However, we are confident that such MIN-related network analyses provide insight into the pathogenesis and/or compensatory mechanisms in various chronic complex diseases. The MIN signature per se could be qualified as a key health index, providing information on the energy supply (deficits) in various diseases. Such analyses open innovative avenues of biomedical research for dissecting complex diseases, with primary or secondary bioenergetic deficiencies. Finally, this approach may well be applicable to the network analysis of other cellular organelles, such as endoplasmic reticula or lysosomes.

## Methods

### Reprogramming of human fibroblasts into iPSCs

We complied with all the relevant ethic regulations and Luxembourg CNER (Comité National d’Ethique de Recherche) has approved the usage of the iPSCs derived from PD patients and the related controls (201411/05). All the participants have provided written informed consent to take part in this study. Both the *SNCA*-mutated patient (p.A30P)^20^ and the unaffected control (Control 1) were 67-year-old male (as a control already used in another study^45^) when the biopsies were collected (Supplementary Table 1). The *SNCA*-triplication patient and the unaffected first-degree relative control (Control 5) were 54- and 34-year-old female, respectively, when the sampling was executed. The *RHOT1*-mutated patient was from the existing German PD cohort (average age of onset of 59.4 ± 13.2 years, average age of sample collection of 65.7 ± 10.2 years). Informed consent was obtained from these patients and controls and approved by the Ethics Committee of the Medical faculty and the University Hospital Tübingen, Germany. The *RHOT1*-mutated late-onset female PD patient (with a heterozygous point mutation c.815 G > A in *RHOT1* [NM_001033568]) had a tremor-dominant clinical phenotype and her father also had tremor in family history. The selected control (Control 2) was age- and gender-matched. More information about the *RHOT1*-mutated patient can be found elsewhere^16^.

Patient dermal fibroblasts carrying the heterozygous p.D620N mutation in *VPS35* were a kind gift from George Mellick from the Griffith Institute (Queensland, Australia). More information on the *VPS35* patient is provide elsewhere^46^. Control fibroblasts from age- and gender-matched healthy individuals 16_33 and 16_1 are from Tübingen’s Biobank. Skin biopsies were performed at the ages of 73, 72, and 77 for *VPS35*-mutated patient, the control 16_33, and the control 16_1, respectively.

Skin fibroblasts of patients or healthy controls were cultured at low-passage number and maintained with Dulbecco’s modified eagle medium (41965-062, Thermo Fisher Scientific) supplemented with 15% fetal bovine serum (10270106, Thermo Fisher Scientific) and 1% penicillin–streptomycin (15140-122, Thermo Fisher Scientific). When confluence was reached, wild-type skin fibroblasts were reprogrammed into induced pluripotent stem cells (iPSCs) via lentivirus infection^47^ using the CytoTune-iPS 2.0 Sendai Reprogramming Kit (A16517, Thermo Fisher Scientific) and patient-derived fibroblasts were reprogrammed into iPSCs via synthetic modified mRNA^46,48^. For the samples derived from control 16_1, the fibroblasts were reprogrammed using the three plasmids (pCXLE-hOct3/4 [Addgene #27076], pCXLE-hSK [Addgene #27078], and pCXLE-hUL [Addgene #27080]) with 10ug of each plasmid through the Amaxa Nucleofector (Lonza). The fibroblasts from other donors were reprogrammed using Sendai virus.

iPSC clones were expanded in culture and maintained with Essential 8 medium (A1517001, Thermo Fisher Scientific) supplemented with 1% penicillin–streptomycin. Cell culture conditions for the maintenance and passaging of the iPSCs have been described elsewhere^46^. Chosen iPSC clones for neuronal differentiation were selected via karyotype analysis and iPSC-characterization procedures^49^.

### Midbrain dopaminergic neuronal differentiation

Following the procedures above, human iPSCs derived from patients or age- and gender-matched healthy controls were obtained and submitted to neuronal differentiation (for details see below). We included human iPSCs from a monogenic, heterozygous dominant familial case of PD, with a point mutation in the *alpha-synuclein* (*SNCA*) gene (Patient 1), from a healthy control (Control 1 that has already been used in another work^45^), and from a patient isogenic control (Patient 1 + mutation correction)^20^. A PD patient with *SNCA* triplication and the corresponding unaffected matched family control (Control 5) were also included in our analysis. The patient isogenic control was obtained by the CRISPR/CAS9-based genome editing to correct the p.A30P-point mutation of *SNCA* found in the Patient 1 case. The detailed method was described elsewhere^20^. We also generated human iPSCs from a genetic PD patient with a point mutation in *RHOT1* (Patient 2)^50^, from a matched healthy control (Control 2), from a familial genetic PD patient with a point mutation in *VPS35*, and from two matched healthy controls (Control 16_33 and 16_1)^46^.

Chosen iPSC clones were differentiated into small-molecule neural progenitor cells (smNPCs) via small molecules of human neural progenitors^51^. Successfully differentiated smNPCs were expanded in culture and maintained with N2B27 medium consisting of 50:50 Neurobasal (21103-049, Thermo Fisher Scientific)/DMEM-F12 (11320-033, Thermo Fisher Scientific) supplemented with 1:200 N2 (17502-048, Thermo Fisher Scientific), 1:100 B27 (17504-044, Thermo Fisher Scientific), 1% glutamax (35050-061, Thermo Fisher Scientific), and 1% penicillin–streptomycin. Dopaminergic neuronal differentiation of smNPCs was performed using the methodology explained elsewhere^51^. Of note, the cells derived from the *VPS35*-mutated patient and the matched controls were differentiated in the medium with or without B27 supplements (as antioxidants), while all the others were cultured with antioxidant supplements.

### Live-cell imaging of iPSC-derived neurons

For the materials derived from the patient with a mutation in *RHOT1* or the related control, neurons at day 25 of maturation were seeded in chamber slides (154534, Thermo Fisher Scientific). Five days were needed for the cells to stabilize in the chamber slides and reach an appropriate level of connectivity. For the materials derived from the patient with a mutation in *VPS35* or the related control, neurons at day 21 of maturation were seeded in chamber slides (154534, Thermo Fisher Scientific). Nine days were needed for the cells to stabilize in the chamber slides and reach an appropriate level of connectivity. For the cells derived from both the *RHOT1*- and the *VPS35*-mutated patient and their corresponding controls, the staining and image analysis is identical. At day 30 of maturation, mDANs were stained for live-cell imaging by using 1:10000 MitoTracker Green FM (M-7514, Thermo Fisher Scientific) to label mitochondria and 1:5000 LysoTracker Deep Red (L-12492, Thermo Fisher Scientific) to label lysosomes. Cellular nuclei were stained with 1:100 Hoechst 33342 (H1399, Thermo Fisher Scientific) after mitochondria and lysosome staining was performed. Neurons were washed once with prewarmed medium prior to imaging. Live-cell imaging was performed using the Live Cell Microscope Axiovert 2000 with spinning disk (Carl Zeiss Microimaging GmbH) in a humidified atmosphere containing 5% CO_2_ at 37 °C.

For the cells derived from the *SNCA*-mutated or -triplication patients or the corresponding controls, the details were slightly different. Neurons at day 35 of differentiation were seeded into coverslips (AB0577, Thermo Fisher Scientific). Ten days were necessary for the cells to stabilize and regenerate the complex network. At day 45 of differentiation, cells were fixed using 4% PFA (J61899.AP, Thermo Fisher Scientific) for 15 min at room-temperature agitating. Permeabilization/blocking was performed using 0.4% Triton X-100 (T8787-100ML, Sigma-Aldrich) in PBS + / + (SH30256.FS, formerly GE Healthcare Europe GmbH, now Cytiva) with 10% goat serum (S26-100ML, Merck Millipore) and 2% BSA (B9000S, New England Biolabs) for 1 h at room temperature. Primary and secondary antibodies were prepared in a solution of 0.1% Triton X-100 (T8787-100ML, Sigma-Aldrich) in PBS + / + (SH30256.FS, formerly GE Healthcare Europe GmbH, now Cytiva) with 1% goat serum (S26-100ML, Merck Millipore) and 0.2% BSA (B9000S, New England Biolabs). For mitochondria detection, we used the Tom20 (sc-11415, Santa Cruz) antibody at 1:500 dilution overnight at 4 °C with agitation. Tom20 was detected by the use of the secondary ab Alexa Fluor^®^ 488 (A-11008, Thermo Fisher Scientific) at 1:1000 dilution, incubated for 3 h at room temperature with agitation. For nuclear staining, we used 1:100 Hoechst 33342 (H1399, Thermo Fisher Scientific) for 15 min at room temperature with agitation. Coverslips were mounted on slides using Vectashield (H-1000, LABCONSULT SPRL/Vectorlab) mounting solution and sealed. Imaging was performed using the Live Cell Microscope Axiovert 2000 with spinning disk (Carl Zeiss Microimaging GmbH) using a 63× objective. For each condition, it was observed that ten nonempty fields were randomly selected, each of them as a *Z stack*, using a 0.2-µm *Z*-axis step and the total number of slices enough to cover the entire depth of the sample. All files were saved for further analysis as .czi files.

### MIN matrix construction

Adjacency matrices of the mitochondria interaction networks (MINs) were extracted from confocal three-dimensional (3D, with Z stack) mitochondrial immunofluorescence images of colonic ganglia^13^, according to a reported method that has been optimized for image-based network analysis^52^. In classical adjacency matrices (*A*) of undirected graphs, the element *A*_*i,j*_ = 1 indicates that there is a link between nodes (mitochondrial branch point) *i* and *j*, *A*_*i,j*_ = 0 otherwise. In contrast to the classical matrix, in the adjacency matrix variant defined by Kerschnitzki et al.^52^, the matrix element *A*_*i,j*_ represents the count of pixels in the link connecting the given nodes *i* and *j* if there is a link and otherwise sets to zero. The key *Matlab* functions for mask skeletonization and adjacency matrix extraction, namely “Skeleton3D” and “Skel2Graph3D,” were kindly provided by the authors of the previous work^52^. For the parameter “THR” of the function “Skel2Graph3D,” defining the minimum length of branches (edges that do not end at another node), to filter out potential skeletonization artifacts, in our analysis, we set as zero to avoid losing any information. Accordingly, in the following network analysis, we considered the existence of a link between the nodes *i* and *j* if *A*_*i,j*_ is larger than zero. The other criteria, masks and filters used for mitochondrial segmentation and pixel calculation were described in our previous work^13^.

### Subnetwork/component extraction and network analysis

The MINs, reconstructed as aforementioned, are potentially disconnected, i.e., they may not form a path between all pairs of nodes. In order to ensure a meaningful calculation of all the analyzed topological metrics, we have proceeded to dissect each MIN into a collection of connected subnetworks/components, thus representing a set of locally interacting mitochondria.

The following standard metrics have been evaluated on the obtained subnetworks:

- Normalized degree (k*). Considering the varying sizes of distinct network components and to make the degree comparable, we normalized the connection degrees of the given nodes within each component using the equation (eq.) $k^ \ast = \frac{k}{N}$ Eq. (1), where *k* is the raw connection degree and *N* is the number of nodes in the given component/subnetwork.
- *Link density and max degree.* Respectively defined as the number of active links over the total number of possible links $( {l_d = \frac{1}{{N(N - 1)}}\mathop {\sum}\nolimits_{i,j \ne i} {a_{i,j}} })$ Eq. (2), and the number of direct connections of the most connected node^7,24^ $\left( {m_d = max_ik_i} \right)$ Eq. (3).
- *ASPL.* The average shortest path length (ASPL) is defined as the average length of the shortest (or geodesic) paths connecting all possible pairs of nodes^24^, i.e., $ASPL = \frac{1}{{N(N - 1)}}\mathop {\sum}\nolimits_{i,j \ne i} {d_{i,j}}$ Eq. (4).
- *Diameter.* Defined as the greatest shortest path length between all pairs of nodes in the networks^24^.
- *Efficiency*. Metric assessing how efficiently information can be transmitted among nodes; it is defined as the harmonic mean of the geodesic distance between all pairs of nodes: $E = \frac{1}{{N(N - 1)}}\mathop {\sum}\nolimits_{i,j \ne i} {\frac{1}{{d_{i,j}}}}$ Eq. (5), *N* being the number of nodes composing the network, and *d*_*i, j*_ the distance (in terms of the number of links) between nodes *i* and *j*^53^.
- *Modularity*. Measuring how much the network is organized into communities, i.e., groups of nodes strongly connected between them but loosely connected with the other nodes of the network^54,55^. The community structure has been detected through the Louvain algorithm^56^.
- *Assortativity*. Pearson’s correlation coefficient between the degrees of both nodes of a link. Positive values indicate that highly connected nodes prefer to link with other hubs, while negative values designate that highly connected nodes prefer to link with periphery nodes^37^.
- *Transitivity*. Density of triangles (triplets of completely connected nodes) in the network.
- *Information content*. The measure of assessing the presence of mesoscale structures, e.g., communities, based on the identification of regular patterns in the adjacency matrix of the network, and on the calculation of the quantity of information lost when pairs of nodes are iteratively merged^26^.
- *Small worldness*. Metric assessing the coexistence of a high clustering coefficient and a low mean geodesic distance^25,57–59^.
- *Motifs*. Specific connectivity patterns, created by a small number of nodes, that exist more frequently in the given networks than in randomized networks^29^. Motifs with three or four nodes have been considered here. We displayed the components with a size ranging from 6 to 28 (Fig. 2d, e). The component with 29 nodes only appeared once in the healthy controls. More precisely, only nine components with size equal to or greater than 29 have been detected for control subjects (representing 0.0471% of the components), and 46 counterparts for PD patients (representing 0.115%). Due to their low frequency and thus low statistical power, when showing the average scores, we did not include very large components (size > = 29) in Fig. 2d, e. This restriction in sizes does not apply to the classification section of ganglia samples. The same has been applied to the *SNCA*-triplication analysis (only shown until size of 36) as the component with 37 nodes only appeared once in the samples of the corresponding control.
- *Comparison of subnetwork size between groups.* Distribution of network components’ sizes, e.g., the curves in Fig. 1b, has further been modeled through a power law *P(s)~k*^*s*^, where *s* is the component size. The fit has been performed through the Levenberg–Marquardt algorithm, and by disregarding the lower and higher tails of the curves, specifically, the 10% of the lower and higher sizes, to thus focus on the central part of the distribution^60^. Pairs of distributions have been compared for the null hypothesis of sharing the same power-law slope *k* (Figs. 1e and 3c). For that, the two slopes have been represented by two normally-distributed random variables, centered on the estimated value of *k* and with a standard deviation equal to the width of the 68% confidence interval of *k*. Finally, the probability of the difference between the means of both distributions of being zero is calculated and converted to a *P* value. Note that the other fits may be compatible, e.g., a cutoff power law^60^; these have not been considered due to the limited availability of very large network components and our interest in the central part of the distribution.

### Normalization through random networks

In order to normalize the values obtained for the listed metrics, a set of 100 random networks were generated for each component, and used as a null model. Each one of these randomized networks is composed of the same number of nodes and links as the original network; additionally, to ensure a biological plausibility, each generated random network was used only if all the nodes and links form a single component. Afterward, each metric is normalized through a *Z* score, calculated as $Z - score = \frac{{M - \mu (M_R)}}{{\sigma (M_R)}}$ Eq. (6), *M* being the value obtained in the real network, *M*_*R*_ the set of values obtained for the random set, and *μ*(·) and σ(·), respectively the average and standard deviation operators.

*Probability of overall* ***Z*** *scores*: For any of the analyzed metrics, the probability of a given *Z* score is defined as follows: $\frac{m}{n}$ Eq. (7), where *m* is number of components/subnetworks with the given *Z* score and *n* is the number of total components within the MINs.

### Classification

The classification models’ performance has been corrected against overfitting by using a modified Leave-One-Out Cross Validation (LOOCV) approach. The standard LOOCV technique entails testing each instance of the data set with a model trained with all other instances, followed by calculating the average classification error. It is worth noting that a simple LOOCV would here lead to an overfitting, as each person in the data set is described by multiple instances (i.e., different neurons and mitochondrial networks). False conclusions may be drawn when using a model trained from the MINs of one neuron for testing another neuron of the same participant. In order to avoid this pitfall, we here employed a modified approach in which each model was trained using the data from all the other people, except from those records belonging to the tested participant. The overfitting issue was also minimized by the fact that we had many more network components/subnetworks than the selected features. Furthermore, we also randomly reshuffled the sample labels 50 times to test whether the high AUC values we achieved in the real datasets can be also obtained even in the randomized datasets.

### Quantification and statistical analysis

We employed the two-sample two-sided *K–S* (Kolmogorov–Smirnov) test in general. However, for the comparison of the exponential fits (Figs. 1e and 3c), we used a different test as a *K–S* test would not work well with a distribution with such a long tail (for details see the description above). Asterisk indicates a significant *P* value (<=0.05) after Šidák correction (the displayed *P* values in the corresponding figures are before correction). Whenever the corresponding test was used, it was directly indicated in the corresponding figure legend. The number of analyzed samples was directly indicated either in Fig. 1 or Fig. 3.

### Reporting summary

Further information on research design is available in the Nature Research Reporting Summary linked to this article.

## Supplementary information

Supplementary Information

Reporting Summary

## Data Availability

All the raw 3D image datasets used in this work with a volume of ~700 G are deposited online in the R3 lab of the University of Luxembourg (https://webdav-r3lab.uni.lu/public/MitoNetworks/).

## Code availability

The computational script codes are accessible at Github (https://github.com/FengHe001/Mitochondria-network-analysis; https://github.com/FengHe001/Network-matrix-extraction). Network adjacency matrix was extracted using *Matlab* codes and network analysis was performed using *Python* scripts.
