# Author Correction: Mitochondria interaction networks show altered topological patterns in Parkinson’s disease

**DOI:** 10.1038/s41540-025-00552-8

**Published:** 2025-07-03

**Authors:** Massimiliano Zanin, Bruno F. R. Santos, Paul M. A. Antony, Clara Berenguer-Escuder, Simone B. Larsen, Zoé Hanss, Peter A. Barbuti, Aidos S. Baumuratov, Dajana Grossmann, Christophe M. Capelle, Joseph Weber, Rudi Balling, Markus Ollert, Rejko Krüger, Nico J. Diederich, Feng Q. HeFeng

**Affiliations:** 1 Instituto de Física Interdisciplinar y Sistemas Complejos IFISC (UIB-CSIC), E-07122 Palma de Mallorca, Spain; 2 Center for Biomedical Technology, Universidad Politécnica de Madrid, Campus of Montegancedo, E-28223 Pozuelo de Alarcón, Madrid, Spain; 3 Luxembourg Centre for Systems Biomedicine (LCSB), University of Luxembourg, Campus Belval, 6, Avenue du Swing, L-4367 Belvaux, Luxembourg; 4 Transversal Translational Medicine, Luxembourg Institute of Health (LIH), 1A-B, rue Thomas Edison, L-1445 Strassen, Luxembourg; 5 Disease Modeling and Screening Platform (DMSP), Luxembourg Institute of Systems Biomedicine, University of Luxembourg & Luxembourg Institute of Health, 6 avenue du Swing, L-4367 Belvaux, Luxembourg; 6 Department of Infection and Immunity, Luxembourg Institute of Health (LIH), 29, rue Henri Koch, L-4354 Esch-sur-Alzette, Luxembourg; 7 Centre Hospitalier de Luxembourg (CHL) 4, Rue Nicolas Ernest Barblé, L-1210 Luxembourg, Luxembourg; 8 Department of Dermatology and Allergy Center, Odense Research Center for Anaphylaxis (ORCA), University of Southern Denmark, 5000C Odense, Denmark; 9 Institute of Medical Microbiology, University Hospital Essen, University Duisburg-Essen, D-45122 Essen, Germany

Correction to: *npj Systems Biology and Applications* 10.1038/s41540-020-00156-4, published online 10 November 2020

In this article the author name Feng Q. HeFeng was incorrectly written as Feng Q. He. The original article has been corrected.

