# Supplementary material for: Mitochondria interaction networks show altered topological patterns in Parkinson’s disease
Source: NPJ Syst Biol Appl. 2020 Nov 10;6:38. doi: 10.1038/s41540-020-00156-4 (PMC7655803; doi:10.1038/s41540-020-00156-4)
Supplement: Supplementary file 1 — Supplementary Information [file 41540_2020_156_MOESM1_ESM.docx]

## **Mitochondria interaction networks show altered topological patterns in Parkinson’s disease**

Massimiliano Zanin^1,2^, Bruno F. R. Santos^3,4^, Paul M.A. Antony^3^, Clara Berenguer-Escuder^3^, Simone B. Larsen^3^, Zoé Hanss^3^, Peter A. Barbuti^3,4^, Aidos S. Baumuratov^3^, Dajana Grossmann^3^, Christophe Capelle^5^, Joseph Weber^6^, Rudi Balling^3^, Markus Ollert^5,7^, Rejko Krüger^3,4,6^, Nico J. Diederich^6^, Feng Q. HeFeng^3,5,8*^

^1^, Instituto de Física Interdisciplinar y Sistemas Complejos IFISC (UIB-CSIC), E-07122 Palma de Mallorca, Spain.

^2^, Center for Biomedical Technology, Universidad Politécnica de Madrid, Campus of Montegancedo, E-28223 Pozuelo de Alarcón, Madrid, Spain.

^3^, Luxembourg Centre for Systems Biomedicine (LCSB), University of Luxembourg, Campus Belval, 6, Avenue du Swing, L-4367 Belvaux, Luxembourg.

^4^, Transversal Translational Medicine, Luxembourg Institute of Health (LIH), 1A-B, rue Thomas Edison, L-1445 Strassen, Luxembourg

^5^, Department of Infection and Immunity, Luxembourg Institute of Health (LIH), 29, rue Henri Koch, L-4354 Esch-sur-Alzette, Luxembourg.

^6^, Centre Hospitalier de Luxembourg (CHL), 4, Rue Nicolas Ernest Barblé, L-1210 Luxembourg, Luxembourg.

^7^, Department of Dermatology and Allergy Center, Odense Research Center for Anaphylaxis (ORCA), University of Southern Denmark, 5000 C, Odense, Denmark.

^8^, Institute of Medical Microbiology, University Hospital Essen, University Duisburg-Essen, D-45122 Essen, Germany

*Correspondence should be addressed to F.Q.H. ([Feng.he@lih.lu](mailto:Feng.he@lih.lu)).

### **Keywords**

Mitochondria interaction network; network biology; network analysis; scale-free; Parkinson’s disease; neurodegenerative diseases; enteric ganglia; iPSC; Mitochondria; Dopaminergic neurons; machine learning.

### **Running title: Mitochondria interaction networks in PD**

### **Supplementary Figures:**


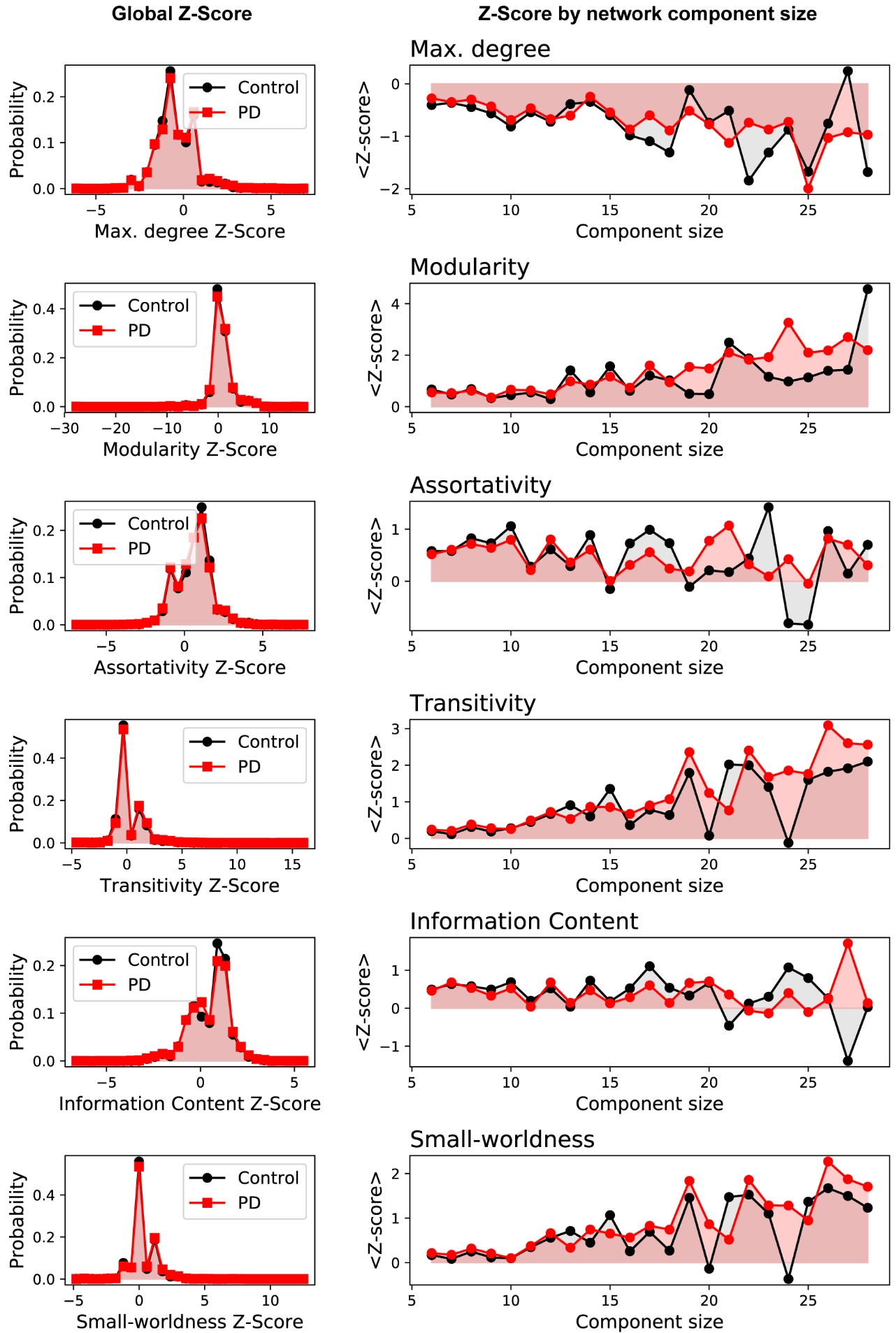


Supplementary Figure 1. Various graph metrics of mitochondria interaction networks (MINs) from enteric ganglia of PD.

Left, probability of different *Z-score* of the global MINs from PD patients or healthy controls were plotted; right, *Z-score* of a given graph metric for the given size of the MIN network components from PD patients or healthy controls. The corresponding statistic results of the graph metrics for the global MINs (refer to **Methods** in main text) were displayed in **Fig.1f**.


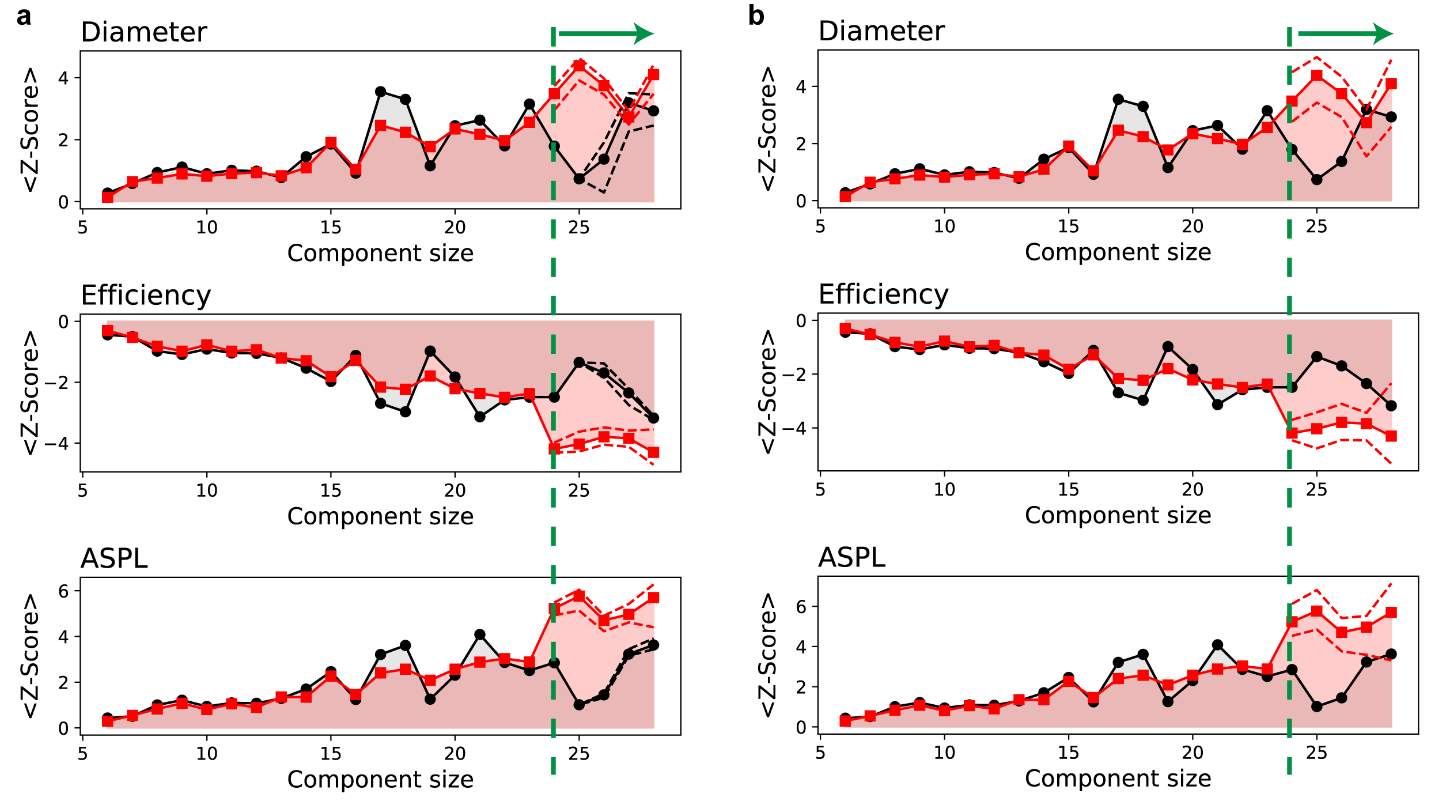


Supplementary Figure 2. Sensitivity analysis in the MINs to the number of subjects.

**a**, The results generated by iteratively deleting one subject in each group. **b**, The graph generated by randomly selecting 4 patients and by repeating 100 times. In a way similar to **Fig. 2a**, black and red lines represent the average *Z-score* for control subjects and patients respectively, as a function of the component size. Additionally, dashed lines represent **a**) the maximum and minimum *Z-score* when one subject was deleted from each group; and **b**) the 15-84% interval when four patients were randomly selected. The red or black lines represent PD or control groups, respectively. The green dashed line and the green arrow above the plots highlight the large components.


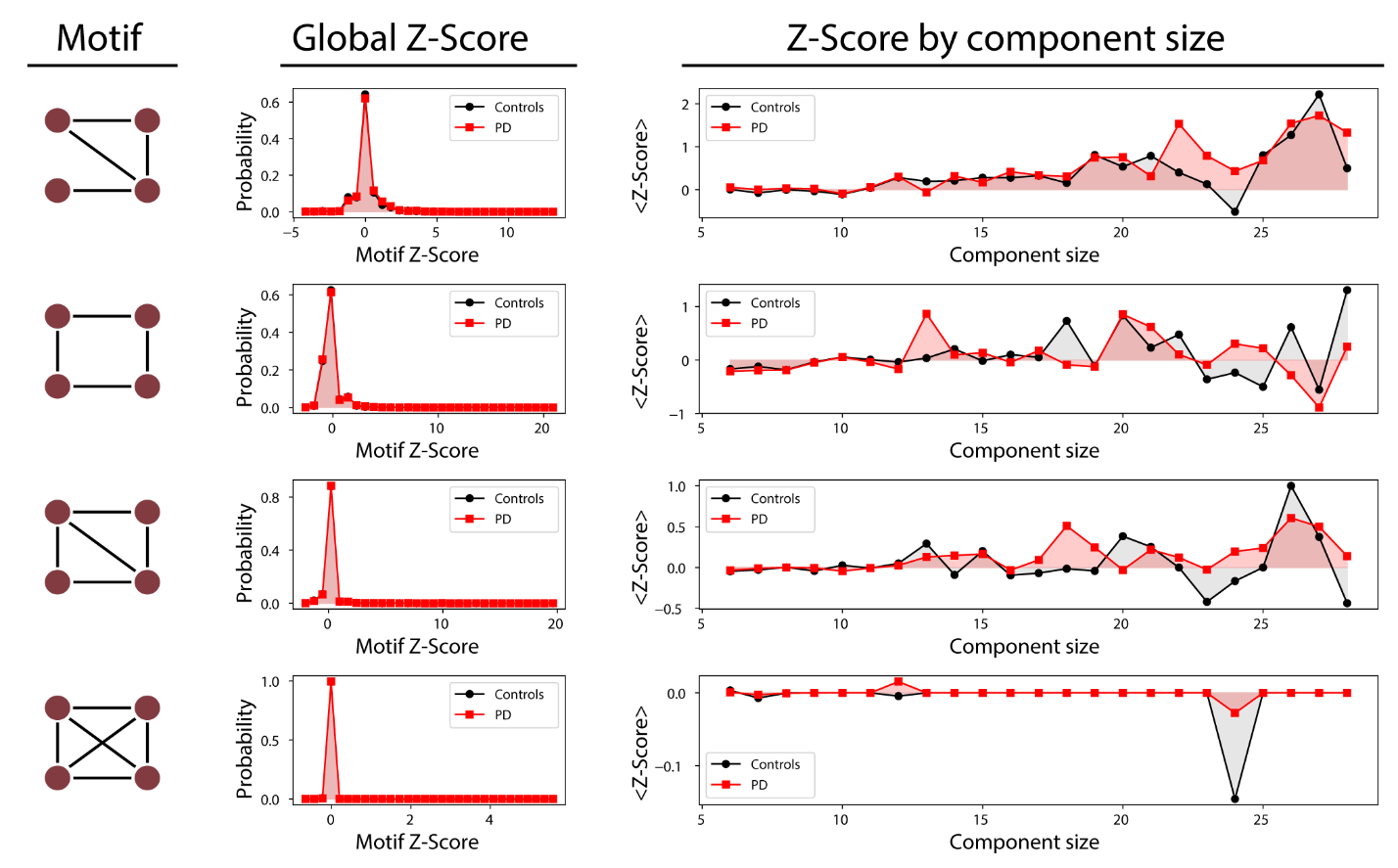


**Supplementary Figure 3. Occurrence of the other types of 4-node motifs within enteric ganglia MINs of PD patients and healthy controls.**

Each row presents information of the particular type of 4-node motifs from PD or healthy controls. The legend of the display is the same as in **Fig. 2e**.


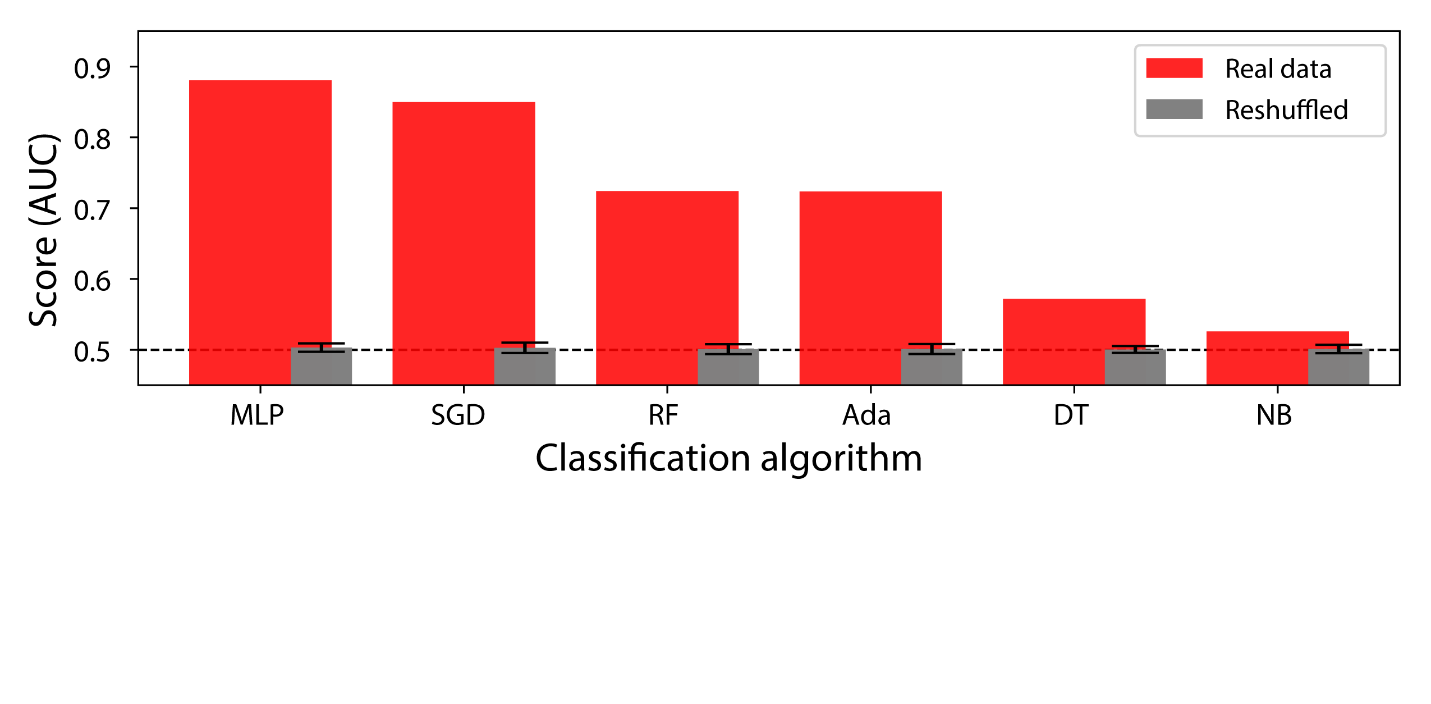


Supplementary Figure 4. Classification performance of different machine-learning approaches using all the MIN features from all the types of enteric ganglia samples vs. randomly-reshuffled datasets.

SGD, Stochastic Gradient Descent; MLP, Multilayer Perception; RF, Random Forest; ADA, Ada-boost; DT, Decision Tree; NB, Naive Bayes. The legend ‘Reshuffled’ indicates that we randomly reshuffled the labels 50 times among our samples to test whether we can obtain similar high AUC results in the randomized datasets. The error bar in reshuffled data represents standard deviation.


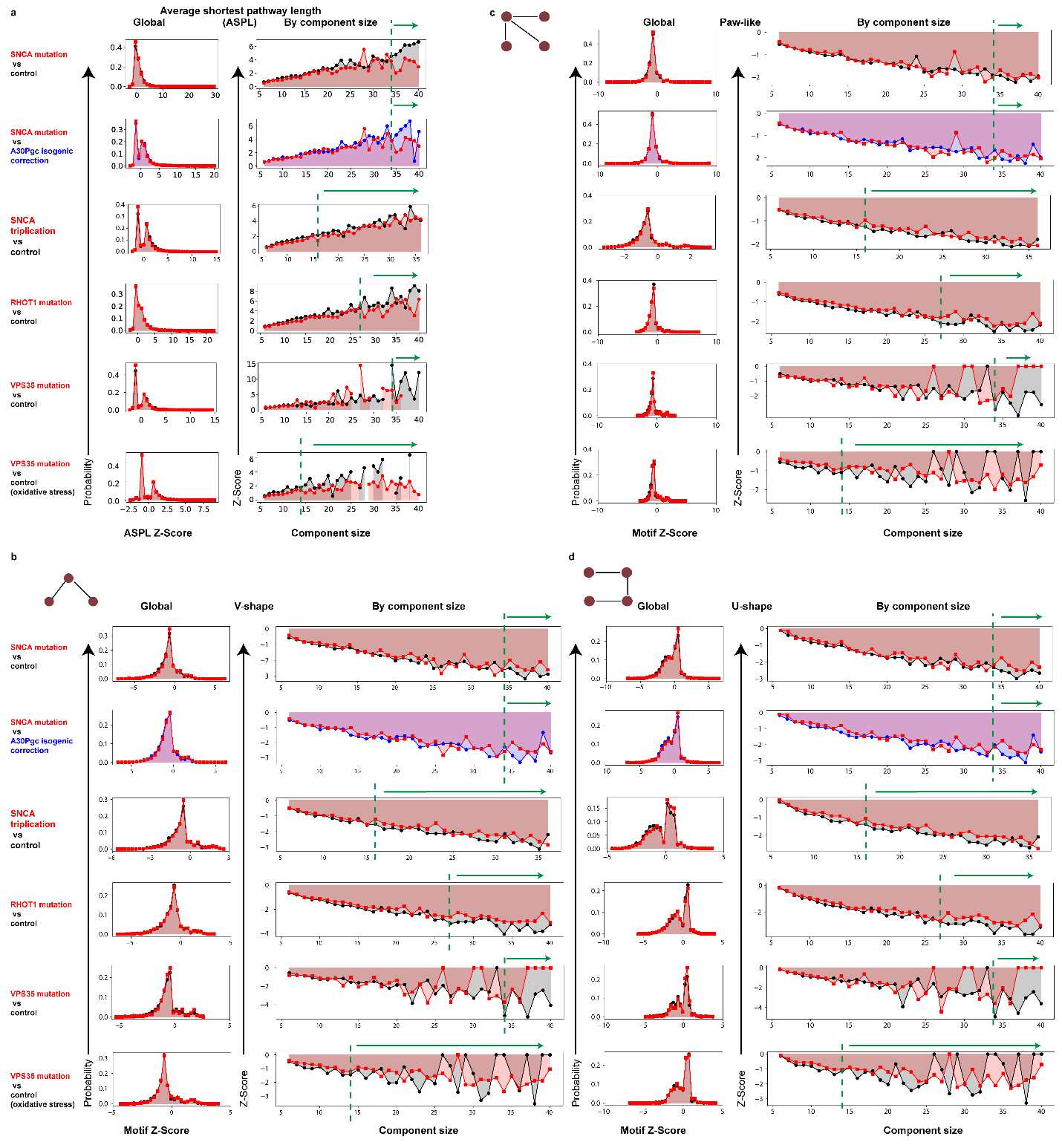


**Supplementary Figure 5. Extended characterization of the MINs of iPSCs-differentiated mDANs of different genetic PD patients.**

**a**, Distribution of *Z-scores* of average shortest pathway length (ASPL). Left, histograms representing the probability distribution of the corresponding *Z-score* from the global MINs of mDANs derived from different patients or matched controls; The right graphs depicting the *Z-scores* for the given size of the MIN components/subnetworks. The green dashed line and the green arrow above the plots highlight the large components that show a clear difference. Of note, mDANs from the *VPS35*-mutated patient and the matched controls were differentiated with or without (w/o) anti-oxidants (as indicated), while the others were all differentiated in the presence of anti-oxidants. **b**, **c**, **d**, Distribution of V-shape motifs (**b**), paw-like 4-node motifs (**c**) and U-shape 4-node motifs (**d**) of the MINs of mDANs derived from different genetic PD patients or matched controls; Left, the histograms representing the probability distribution of the corresponding *Z-scores* of global MINs from different genetic PD patients or matched controls; the right graphs depicting the *Z-scores* for various sizes of the network components. The green dashed line and the green arrow above the plots highlight the large components that show a clear difference. Since the component with 37 nodes only appeared once in the corresponding healthy controls matching the *SNCA*-triplication patient, we did not show very large components (size>=37) in the analysis.

### **Supplementary Tables**

Supplementary Table 1. Information related to PD patients and the matched controls used in this study.

| **ID information** | **No. of patients** | **No. of Controls** | **Gender** | **Age of biopsy** | **Purposes** | **Mutation** | **Fibroblast ID** | **Resource Institution** | **Related references** |
| --- | --- | --- | --- | --- | --- | --- | --- | --- | --- |
| SNCA | 1 | 0 | male | 67 | PD patient | c.88G>C, *SNCA* p.A30P |  | LCSB | Ref ^1-3^ |
| Control 1 | 0 | 1 | male | 67 | Control |  | 17608 | Tübingen biobank | Ref ^4^ |
| SNCA GC | 0 | 1 | male | 67 | Gene correction of the *SNCA* p.A30P patient |  |  | LCSB | Ref ^5^ |
| SNCA triplication | 1 | 0 | female | 54 | PD patient | SNCA triplication |  | EBiSC (https://ebisc.org/) | N.A. |
| Control 5 | 0 | 1 | female | 34 | Patient’s immediate family control |  |  | EBiSC  (https://ebisc.org/) | N.A. |
| RHOT1 | 1 | 0 | female | 78 | PD patient | c.815G>A, *RHOT1* p.R272Q |  | LCSB | Ref ^6,7^ |
| Control 2 | 0 | 1 | female | 72 | Control |  | 18075 | Tübingen Biobank | Ref ^6,7^ |
| VPS35 | 1 | 0 | male | 73 | PD patient | c.1858G>A *VPS35* p.D620N |  | Griffith Institute (Queensland, Australia) | Ref ^8^ |
| Control 3 [16426 (16_33)] | 0 | 1 | male | 72 | Control |  | 16426 | Tübingen Biobank | N.A. |
| Control 4 [16535 (16_1)] | 0 | 1 | male | 77 | Control |  | 16535 | Tübingen Biobank | N.A. |
| iPD | 11 | 0 | 5 male; 6 female | 70±6 | Colon ganglia analysis of idiopathic PD |  |  | Luxembourg | Ref ^9^ |
| controls | 0 | 4 | 1 male; 3 female | 65±5 | Colon ganglia analysis of Healthy controls (recruited for idiopathic PD) |  |  | Luxembourg | Ref ^9^ |
| **Total number** | **15** | **10** |  |  |  |  |  |  |  |

N.A., References not available yet.

### **References**

1 Kruger, R. *et al.* Ala30Pro mutation in the gene encoding alpha-synuclein in Parkinson's disease. *Nat Genet* **18**, 106-108, doi:10.1038/ng0298-106 (1998).

2 Kruger, R. *et al.* Familial parkinsonism with synuclein pathology: clinical and PET studies of A30P mutation carriers. *Neurology* **56**, 1355-1362, doi:10.1212/wnl.56.10.1355 (2001).

3 Barbuti, P. A. *et al.* Generation of two iPS cell lines (HIHDNDi001-A and HIHDNDi001-B) from a Parkinson’s disease patient carrying the heterozygous p.A30P mutation in SNCA. *Stem Cell Research* **48**, 101951, doi:<https://doi.org/10.1016/j.scr.2020.101951> (2020).

4 Schondorf, D. C. *et al.* iPSC-derived neurons from GBA1-associated Parkinson's disease patients show autophagic defects and impaired calcium homeostasis. *Nat Commun* **5**, 4028, doi:10.1038/ncomms5028 (2014).

5 Barbuti, P. *et al.* Using High-Content Screening to Generate Single-Cell Gene-Corrected Patient-Derived iPS Clones Reveals Excess Alpha-Synuclein with Familial Parkinson’s Disease Point Mutation A30P. *Cells* **9**, 2065 (2020).

6 Berenguer-Escuder, C. *et al.* Impaired mitochondrial-endoplasmic reticulum interaction and mitophagy in Miro1-mutant neurons in Parkinson's disease. *Hum Mol Genet* **29**, 1353-1364, doi:10.1093/hmg/ddaa066 (2020).

7 Grossmann, D. *et al.* Mutations in RHOT1 Disrupt Endoplasmic Reticulum-Mitochondria Contact Sites Interfering with Calcium Homeostasis and Mitochondrial Dynamics in Parkinson's Disease. *Antioxid Redox Signal* **31**, 1213-1234, doi:10.1089/ars.2018.7718 (2019).

8 Larsen, S. B. *et al.* Induced pluripotent stem cell line (LCSBi001-A) derived from a patient with Parkinson's disease carrying the p.D620N mutation in VPS35. *Stem Cell Res* **45**, 101776, doi:10.1016/j.scr.2020.101776 (2020).

9 Baumuratov, A. S. *et al.* Enteric neurons from Parkinson's disease patients display ex vivo aberrations in mitochondrial structure. *Sci Rep* **6**, 33117, doi:10.1038/srep33117 (2016).
