# Supplementary material for: Mitochondria interaction networks show altered topological patterns in Parkinson’s disease
Source: NPJ Syst Biol Appl. 2020 Nov 10;6:38. doi: 10.1038/s41540-020-00156-4 (PMC7655803; doi:10.1038/s41540-020-00156-4)
Supplement: Supplementary file 2 — Reporting Summary [file 41540_2020_156_MOESM2_ESM.pdf]

nature research

Corresponding author(s): Feng He

Last updated by author(s): Sep 12, 2020

Reporting Summary

Nature Research wishes to improve the reproducibility of the work that we publish. This form provides structure for consistency and transparency in reporting. For further information on Nature Research policies, see our Editorial Policies and the Editorial Policy Checklist.

Statistics

For all statistical analyses, confirm that the following items are present in the figure legend, table legend, main text, or Methods section.

n/a	Confirmed	
☐	☒	The exact sample size (n) for each experimental group/condition, given as a discrete number and unit of measurement
☐	☒	A statement on whether measurements were taken from distinct samples or whether the same sample was measured repeatedly
☐	☒	The statistical test(s) used AND whether they are one- or two-sided<br>*Only common tests should be described solely by name; describe more complex techniques in the Methods section.*
☒	☐	A description of all covariates tested
☐	☒	A description of any assumptions or corrections, such as tests of normality and adjustment for multiple comparisons
☐	☒	A full description of the statistical parameters including central tendency (e.g. means) or other basic estimates (e.g. regression coefficient) AND variation (e.g. standard deviation) or associated estimates of uncertainty (e.g. confidence intervals)
☐	☒	For null hypothesis testing, the test statistic (e.g. F, t, r) with confidence intervals, effect sizes, degrees of freedom and P value noted<br>*Give P values as exact values whenever suitable.*
☒	☐	For Bayesian analysis, information on the choice of priors and Markov chain Monte Carlo settings
☒	☐	For hierarchical and complex designs, identification of the appropriate level for tests and full reporting of outcomes
☐	☒	Estimates of effect sizes (e.g. Cohen's d, Pearson's r), indicating how they were calculated

Our web collection on statistics for biologists contains articles on many of the points above.

Software and code

Policy information about availability of computer code

Data collection	Live-cell imaging was performed using the Live Cell Microscope Axiovert 2000 with spinning disc (Carl Zeiss Microimaging GmbH) using a 63x objective. For each condition, it was acquired ten non-empty fields randomly selected, each of them as a Z-stack, using a 0.2 µm Z-axis step and a total number of slices enough to cover the entire depth of the sample.
Data analysis	The computational script codes are accessible at Github (https://github.com/FengHe001/Mitochondria-network-analysis; https://github.com/FengHe001/Network-matrix-extraction). Network adjacency matrix was extracted using Matlab codes and network analysis was performed using Python scripts.

For manuscripts utilizing custom algorithms or software that are central to the research but not yet described in published literature, software must be made available to editors and reviewers. We strongly encourage code deposition in a community repository (e.g. GitHub). See the Nature Research guidelines for submitting code & software for further information.

Data

Policy information about availability of data

All manuscripts must include a data availability statement. This statement should provide the following information, where applicable:

- Accession codes, unique identifiers, or web links for publicly available datasets
- A list of figures that have associated raw data
- A description of any restrictions on data availability

Raw 3-D image datasets (with a volume of 691 Gigabytes) are deposited online through the R3 lab of University of Luxembourg (https://webdav-r3lab.uni.lu/public/MitoNetworks/). The files are too huge to be easily deposited in another public server.

Field-specific reporting

Please select the one below that is the best fit for your research. If you are not sure, read the appropriate sections before making your selection.

☒ Life sciences ☐ Behavioural & social sciences ☐ Ecological, evolutionary & environmental sciences

For a reference copy of the document with all sections, see nature.com/documents/nr-reporting-summary-flat.pdf

Life sciences study design

All studies must disclose on these points even when the disclosure is negative.

Sample size	No statistical method was used to pre-decide the sample size. The sample size of our study was chosen based on our previous experience in related projects.
Data exclusions	No data was excluded.
Replication	Each sample was imaged around 10 times in different areas of the slides to cover the properties of an entire sample.
Randomization	The areas of the slides were randomly chosen for different slides.
Blinding	The computational analysis was blind to the biological groups before showing the final results to the senior authors. For the ganglia samples, the experimental operators were not aware of the status of the subjects in advance. It is not possible for the iPSC experimental operators to be blind to the cell groups as they generated and analyzed the cell lines.

Reporting for specific materials, systems and methods

We require information from authors about some types of materials, experimental systems and methods used in many studies. Here, indicate whether each material, system or method listed is relevant to your study. If you are not sure if a list item applies to your research, read the appropriate section before selecting a response.

Materials & experimental systems

n/a	Involved in the study	
☐	☒	Antibodies
☒	☐	Eukaryotic cell lines
☒	☐	Palaeontology and archaeology
☒	☐	Animals and other organisms
☐	☒	Human research participants
☒	☐	Clinical data
☒	☐	Dual use research of concern

Methods

n/a	Involved in the study	
☒	☐	ChIP-seq
☒	☐	Flow cytometry
☒	☐	MRI-based neuroimaging

Antibodies

Antibodies used	ab information is provided in the following order: ab name (cat. nr., provider), dilution factor<br>MitoTracker Green FM (M-7514, Thermo Fisher Scientific) at 1:10000 dilution<br>Tom20 (sc-11415, Santa Cruz) antibody at 1:500 dilution<br>Secondary ab Alexa Fluor® 488 (A-11008, Thermo Fisher Scientific) at 1:1000 dilution,<br>Hoechst 33342 (H1399, Thermo Fisher Scientific) at 1:100 dilution<br>MitoTracker Green FM (M-7514, Thermo Fisher Scientific) at 1:10000 dilution<br>LysoTracker Deep Red (L-12492, Thermo Fisher Scientific) at 1:5000 dilution
Validation	All the antibodies are commercially available and are already validated by the provider and other labs as demonstrated in the reference list of the corresponding antibodies online. The validation information is already provided in the webpage of the provider.

Human research participants

Policy information about studies involving human research participants

Population characteristics	Necessary information of the subject demographics has been provided in Supplementary Table 1 and/or relevant references. Here we just listed some essential information.<br><br>For the iPSCs-derived mDANs analysis of the genetic PD patients, the information is listed in the following order: ID information, Gender, Age of biopsy, genetic characteristic.

April 2020

1 PD patient with mutation in SNCA, male, 67-year old, point mutation c.88G>C, SNCA p.A30P;
The corresponding Control 1, male, 67-year old;
1 SNCA gene correction (GC), male, 67-year old, GC of the SNCA p.A30P patient;
1 SNCA triplication, female, 54-year old PD patient;
1 corresponding family Control 5, female, 34-year old (child of the SNCA triplication PD patient);
1 PD patient with RHOT1 mutation, female, 78-year old, point mutation c.815G>A, RHOT1 p.R272Q ;
1 corresponding Control 2, female, 72-year old;
1 PD patient with VPS35 mutation, male, 73-year old, point mutation c.1858G>A VPS35 p.D620N;
1 corresponding Control 3 [16426 (16_33)] of the VPS35 PD patient, male, 72-year old;
1 corresponding Control 4 [16535 (16_1)] of the VPS35 PD patient, male, 77-year old.

For colon ganglia analysis of 11 sporadic PD patients, 5 male and 6 female, 70±6-year old;
For colon ganglia analysis of 4 healthy controls, 1 male and 3 female, 65±5-year old.

Recruitment

Patients with the genetic form of PD donated their fibroblasts with informed consent. Age- and gender-matched controls were obtained from the Tuebingen biobank or Luxembourg.

For the sporadic PD patients, they did not display any apparent gastrointestinal symptoms at the time of recruitment. Those patients and controls were recruited for colonoscopy analysis. No any bias or self-selection took place during the recruitment. For detailed information related to sporadic PD patients, please refer to our previous work (Baumuratov et al., 2016).

Ethics oversight

Ethical approval for the development of and research pertaining to patient-derived cell lines have been given by informed consent for the academic research project (CNER #201,411/05): “Disease modeling of Parkinson's disease using patient-derived fibroblasts and induced pluripotent stem cells” (DiMo-PD).

Note that full information on the approval of the study protocol must also be provided in the manuscript.
